# Microbial Carotenoid Synthesis Optimization in Goat Cheese Whey Using the Robust Taguchi Method: A Sustainable Approach to Help Tackle Vitamin A Deficiency

**DOI:** 10.3390/foods12030658

**Published:** 2023-02-03

**Authors:** Luis Carlos Mata-Gómez, Paula Mapelli-Brahm, Antonio J. Meléndez-Martínez, Alejandro Méndez-Zavala, Lourdes Morales-Oyervides, Julio Montañez

**Affiliations:** 1 Facultad de Ciencias Quimicas, Universidad Autonoma de Coahuila, Unidad Saltillo, Saltillo 25280, Coahuila, Mexico; 2 Food Colour and Quality Laboratory, Facultad de Farmacia, Universidad de Sevilla, 41012 Sevilla, Spain

**Keywords:** Taguchi, biotechnology, carotenoids, yeast, provitamin A, sustainability

## Abstract

The work describes the carotenoid synthesis process by *Rhodotorula glutinis* P4M422 using an agro-industrial waste as the substrate, seeking a biorefinery platform approach for waste utilization to produce high-value molecules. A culture medium based on goat milk whey (GMW) was optimized via the Taguchi method (L9 array). Four factors (ethanol, carbon and nitrogen source, and pH) were evaluated at three levels. The carbon and nitrogen composition were the factors dominating the process performance. Optimized conditions were validated (Urea, 0.3% *w*/*v*; pH, 4.5; ethanol, 10% *v*/*v*; glucose, 6.0%), and the carotenoid production (4075 µg/L) was almost 200% higher than when using the un-optimized process (2058 µg/L). Provitamin A carotenoids torulene, β–carotene, and γ–carotene (different proportions) were produced under all conditions. The hydrolyzed goat milk whey showed promising expectations as a low-cost source for carotenoid production by *Rhodotorula glutinis* P4M422. The results are important for the innovative sustainable production of carotenoid-rich matrices for different purposes (nutrition, health promotion, color) and industries (foods, nutricosmetics, nutraceuticals, feeds), notably to help to combat vitamin A deficiency.

## 1. Introduction

Carotenoids are non-nitrogenized, highly unsaturated molecules typically containing 40 atoms of carbons with colorant and health-promoting properties [1]. Recently, interest in carotenoids has increased due to the growing demand by industries such as feed additives, pharmaceuticals, aquaculture, food, and cosmetics [2]. Increasing demands on carotenoids have impacted these compounds’ global market (USD 2.0 billion in 2022). According to the most recent Global Market for Carotenoids report [3], this market should reach USD 2.7 billion by 2027, with an annual growth rate of 5.7% (CAGR, 2022–2027).

On the other hand, some carotenoids are precursors of vitamin A, the vitamin with the broadest spectrum of functions in the human body. Indeed, they are key in combating vitamin A deficiency (VAD), one of the most critical public health problems due to nutritional deficiencies, especially in developing countries. Common strategies to combat VAD include supplementation with vitamin A (retinyl acetate) or provitamin A carotenoids (PAC) in supplements or fortified foods, the development of PAC-rich products (via common breeding techniques or genetic engineering) to eventually increase their dietary intake, and the enhancement of post-harvest handling and cooking practices to reduce the loss of PACs [4]. Additionally, the presence of carotenoids in the diet is often associated with health benefits; they could be beneficial in enhancing immune system functions and reducing the risk of developing chronic diseases, including several types of cancer (breast, cervical, ovarian, colorectal), cardiovascular disease, eye conditions, type 2 diabetes, obesity, skin disorders, or metabolic disorders [4]. In addition, in recent years, certain investigations have revealed that carotenoids could act as promoters of cognitive functions [1].

These valuable molecules are widely distributed in nature and can be found in algae, plants (in diverse structures including leaves, fruits, flowers, or roots), animals (invertebrates, fishes, birds, etc.), and microorganisms (bacteria, microalgae, and yeasts). Human beings obtain carotenoids mainly from fruits and vegetables. Some foods rich in carotenoids are tomato, corn, carrots, pumpkin, oranges, and watermelon, among others [1,5,6]. Production of microbial carotenoids presents advantages over that of those extracted from plants or chemically synthesized. For instance, microbial production can be optimized to increase yields in controlled bioreactors [7], eliminating the need for a large production area and seasonal dependency. Additionally, unlike synthetic carotenoids, microorganisms can produce carotenoids of high purity under a biorefinery concept using renewable sources [8].

Concerning red yeasts, such as *Rhodotorula* sp., can produce considerable amounts of carotenoids, such that levels varying from 50 to 350 µg/g dry weight have been reported [9]. The red yeast *Rhodotorula glutinis* can synthesize carotenoids using agro-industrial wastes and/or low-cost substrates [10]. 

Recently, these substrates, such as crude glycerol, cheese whey, sugarcane juice, molasses, mesquite pods extract, corn steep liquor, and straw hydrolysate, among others, have gained attention for the production of carotenoids by different yeasts [11,12,13,14,15,16,17,18]. These industrial by-products contain sugars and other organic compounds, which allow microbial growth and metabolite production. Developing countries such as Mexico possess great potential for using agro-industrial residues as raw materials to synthesize added-value compounds to ensure a bio-based economy due to the large amount of waste generated. For instance, the annual production of goat milk in Mexico has increased in the last decade, reaching over ≈164,000 million liters in 2020 [19]. 

Goat milk can be used for fresh and aged cheeses, sweets, and cream [20]. However, milk processing causes environmental impacts due to the dairy by-products, such as a considerable amount of goat cheese whey generated from cheese production factories [21]. Most local goat milk processing plants do not treat those streams after processing; it is vital to provide alternatives to re-valorize this by-product. Efficient and low-cost processes to minimize waste treatment costs by generating revenues will be potentially profitable for all goat milk cheese plants. Moreover, as recently pointed out, an integrated biorefinery platform for waste utilization to produce high-value and competitive biomolecules is key towards closing the circular bio-economy loop [22,23].

Goat milk whey contains lactose, proteins, and other components that can be converted into pigments, enzymes, biofuels, and organic acids. Carotenoids are molecules with high added value and, thus, with the potential to achieve a cost-effective process. Many researchers have attempted to produce carotenoids using milk whey as a substrate by yeast and fungi [24,25,26,27,28]. However, it has been demonstrated that medium components and process conditions have a strong influence on carotenoid production and biomass growth [17]. Evaluating such factors without a proper method for optimization could be time- and cost-consuming. Increasing demands in the biotechnology industry require process improvement and process understanding. Consequently, the Taguchi method has gained popularity among bioprocess optimization [29] due to its utilization of fractional factorial designs (orthogonal arrays) with minimum experimental requirements for process improvement. Another advantage is that it does not rely on fitting a mathematical model to the experimental data. This method has been applied to efficiently identify the medium components’ influence on the microbial production of carotenoids [14,30]. On the other hand, the synthesized carotenoid’s profile can be affected by the utilized process conditions.

This work aims to optimize the provitamin A carotenoid production in *Rhodotorula glutinis* P4M422 in a medium based on goat milk whey. This objective is totally aligned with key agro-food challenges, as the harnessing of agro-food by-products and waste is essential for the sustainable production of foods, and microbes, along with insects and algae, are among the matrices that can lead to innovations in this context, as pointed out in the recent EAT–Lancet Commission report on healthy diets from sustainable food systems [31]. To the best of our knowledge, the production and characterization of carotenoid’s profile by *Rhodotorula glutinis* in goat milk whey has not been reported. The study results are important in the context of the sustainable production of colorful carotenoid-rich matrices for different purposes (nutrition, health promotion, provision of color) and industries (foods, nutricosmetics, nutraceuticals, feeds), notably to help to combat VAD.

## 2. Materials and Methods

### 2.1. Microorganism and Medium Based on GMW Preparation

*Rhodotorula glutinis* P4M422 was provided by the microbial collection from Food Research Department (FRD, Universidad Autónoma de Coahuila, México). The strain was isolated from the sotol process production plant in Nazas City (Durango, México). The strain was preserved in Petri dishes containing yeast malt (YM) agar at 4 °C (Enriquez and Casas, 2012).

Goat milk whey was obtained from a local goat cheese factory (San Buenaventura, Coahuila, México). Cheese whey was deproteinized by autoclaving (15 min at 120 °C and 15 psi), filtered, and finally micro-filtered (0.45 µm). 

### 2.2. Enzymatic Hydrolysis of Goat Milk Whey

Enzymatic hydrolysis was performed with a commercial enzyme (β–galactosidase, Chr-Hansen, Hoersholm, Denmark) in a ratio of 1:1000 (enzyme: whey) during 120 min at pH 6.5, temperature of 37 °C, and 200 rpm of agitation in an orbital shaker (Inova, New Brunswick Scientific, Edison, NJ, USA). Glucose released from GMW was measured to determine the maximum time of hydrolysis by an enzymatic kit (Gluc-Pap, Randox, Kearneysville, WV, USA). The reducing sugar content was calculated as a function of the glucose released to adjust carbon source concentration in hydrolyzed GMW.

### 2.3. Inoculum Preparation

*Rhodotorula glutinis* P4M422 was propagated in 250 mL Erlenmeyer flasks using 50 mL of YM medium (glucose 1%, yeast extract 0.3%, malt extract 0.3% and peptone 0.5%) during 24 h, and 1 × 10^6^ cell/mL was inoculated in each experimental trial.

### 2.4. Culture Conditions and Experimental Design

Carotenoid production in a low-cost GMW hydrolyzed supplemented with glucose, urea, and ethanol was optimized following the robust engineering methodology of Taguchi [32]. An orthogonal array layout L9 (4^3^) was selected to evaluate the effect of four factors involved in medium composition: glucose (carbon source), urea (nitrogen source), and ethanol supplementation, as well as medium pH at three levels. The variables, their levels, and the combinations tested with the experimental design are given in Table 1.

Each trial was inoculated with 1 × 10^6^ cells/mL, and the experiments were carried out in an orbital shaker (Inova, New Brunswick Scientific, USA) for 72 h at a temperature of 30 °C and 200 rpm. After fermentation, samples were taken to analyze total carotenoid and biomass content.

### 2.5. Spectrophotometric Quantification of Total Carotenoids

Cells were recovered via centrifugation (8000 rpm, 10 min, 4 °C) and washed twice with distilled water. Dimethyl sulfoxide (DSMO, Sigma-Aldrich, Toluca, México) was added to the cells, and the mixture was vortexed; cell lysis was favored by ultrasonication for 15 min. Extractions were carried out until completely colorless cells were obtained. The colored extracts were recovered via centrifugation (8000 rpm, 10 min at 20 °C), and the supernatant was withdrawn. Absorbance was measured in a spectrophotometer UV/VIS (Unico 2150, USA) at 480 nm. The carotenoid concentration was calculated according to Equation (1) [33]:

(1) $$Car=\left(1000*A_{480}\right)/220$$

where *Car* = carotenoid concentration (µg of carotenoids/L) and *A*_480_ = absorbance at 480 nm of wavelength.

### 2.6. Biomass Quantification

Cells were recovered via centrifugation (Hermle Z300 K, Wehingen, Germany) at 8000 rpm for 10 min at 4 °C and washed twice with distilled water. Samples were dried until constant weight at 60 °C for 48 h. 

### 2.7. Analysis of Carotenoids

#### 2.7.1. Sample Preparation

The extractions were carried out until completely colorless cells were obtained. The cells were separated via centrifugation (8000 rpm, 10 min at 20 °C), and the colored supernatant was withdrawn. The cells were washed with acetone (1:1), and the colored extracts were recovered and pooled. Water (1:2) and hexane (1:1) were added to the colored extract in acetone to obtain a concentrated carotenoid-containing extract. The mixture was shaken in a vortex and subsequently centrifuged to separate the phases (8000 rpm, for 15 min at 20 °C). The colored phase was withdrawn and concentrated at 30 °C for 25 min (Eppendorf Concentrator Plus, Eppendorf Ibérica, Spain). The dry carotenoid extracts were re-dissolved in ethyl acetate, micro-filtered (0.45 µm, Millipore) and centrifuged (12,000 rpm, 15 min at 4 °C) prior to the HPLC analysis.

#### 2.7.2. HPLC Analysis

The HPLC analysis was performed on an Agilent 1100 system with a quaternary pump, a photodiode array detector, a column temperature control module, and an auto-sampler (Agilent, Palo Alto, Santa Clara, CA, USA). The sample injection volume was 30 µL. The flow rate was set at 1.0 mL/min. A YMC C30 column (5 µm, 250 × 4.6 mm YMC, Wilmington, NC, USA) kept at 20 °C was used for the separations. A linear gradient method was used with methanol (MeOH), methyl *tert*-butyl ether (MTBE) and water as mobile phase. The gradient elution was 0 min, 70% MeOH + 25% MTBE + 5% water; 7.5 min, 62% MeOH + 38% MTBE; 15 min, 49% MeOH + 51% MTBE; 22.5 min, 36% MeOH + 64% MTBE; 30 min, 23% MeOH + 77% MTBE; 37.5 min, 10% MeOH + 90% MTBE; and finally, at 45 min, 70% MeOH + 25% MTBE + 5% water. The chromatograms were monitored at 450 nm (for β–carotene), 462 nm (for γ–carotene), 484 nm (for torulene), and 286 nm (for phytoene). The identification of carotenoids was performed by comparison of their chromatographic and UV/vis spectroscopic characteristics with those reported in the literature or of standards isolated from appropriate sources according to classical procedures used in our laboratory. Specifically, the β-carotene standard was isolated from carrot roots and the γ-carotene standard from fruits of *Geophila repens.* Torulene, a carotenoid produced by several yeasts, was identified by comparing the data reported elsewhere [34].

### 2.8. Data Analysis

The influence of individual factors on total carotenoid production (TC, µg/L), biomass growth (B, g/L), and yield of carotenoids per biomass (Y, µg/g) was analyzed using Qualitek-4 software (Nutek Inc., Bloomfield Hills, MI, USA). The automatic design option allows Qualitek-4 to select the array and assign factors to the appropriate columns. 

## 3. Results

### 3.1. Enzymatic Hydrolysis of Goat Milk Whey

Hydrolyzed goat milk whey was evaluated as a substrate to develop a low-cost process for carotenoid production. The maximum glucose yield (13 g/L) during the enzymatic hydrolysis of goat cheese whey was reached after 90 min of hydrolysis. However, no significant yield increase (*p* ˂ 0.05) was observed between 90 and 120 min (data not shown). Thus, 90 min was selected as hydrolysis time for further experiments. Similarly, Valduga et al. (2009) reported a maximum glucose–galactose yield at 90 min during the enzymatic hydrolysis of cheese whey; however, they reported a higher sugar release (21.0 g/L). The difference could be attributed to the lactose content of goat cheese whey (10–15 g/L) [35].

For years, some techniques to conduct lactose hydrolysis have been developed to improve the yields of glucose delivery. A disrupted dairy culture for lactose hydrolysis has been previously evaluated [36], where it was reported that the produced biomass and disrupted from 1 L of culture medium was able to hydrolyze the 60% of lactose present in 1 L of milk in 96 h, this being a marked disadvantage for the process developed. Otherwise, Valduga et al. (2009) evaluated the effect of chemical and enzymatic hydrolysis on glucose delivery of cheese whey. They reported that the enzyme’s effectiveness was higher than that of acid hydrolysis due to the degradation of glucose owed to Maillard reactions occurring at high temperatures. 

### 3.2. Optimization by Taguchi Method

The impact of each factor on carotenoid production, biomass growth, and the yield of carotenoids per biomass was evaluated using a Taguchi design, yet only total carotenoid production was considered the target response. Table 2 shows all response variables’ results for each trial, where it can be observed that the responses varied in all trials. 

The carotenoid production ranged from 739.39 ± 41.99 to 3406.06 ± 75.70 µg/L (~4–6-fold difference), the biomass varied from 4.67 ± 0.58 to 15.67 ± 0.29 g/L (~3.4-fold difference), and the carotenoids per biomass varied from 146.33 ± 8.17 to 229.75 ± 8.79 µg/g (~1.6-fold difference). Interestingly, lower production of carotenoids and biomass growth and higher production and growth were obtained in the same trials (Trial 9 and 3, respectively). Meanwhile, the minimum and maximum yields were obtained under different conditions (Trial 8 and 9). This indicates that even though production and growth seem to vary similarly, production per biomass is affected differently. 

The above is confirmed by the analysis of variance (ANOVA) shown in Table 3. The ANOVA describes the actual contribution of each factor and if it is statistically significant. *F* ratio values above 3.00 were significant at 90% of the significance level. 

It can be seen that for P and B, the error is minimum, which indicates that the evaluated factors dominate the performance of these two responses. On the other hand, for Y, the error is 16.4%, meaning that there are noise factors interactions between factors that were not quantified. 

It is also important to note that almost all the factors under the evaluated levels were significant (F > 3.00) on carotenoid production, growth, and yield by *Rhodotorula glutinis* on hydrolyzed goat milk whey. As assumed by looking at the data in Table 2, the ANOVA confirmed that the factors similarly affect the production of pigments and microorganism growth. This phenomenon could be expected because the evaluated factors are involved in microbial growth and carotenoids are highly related to biomass production.

**Figure 1:**
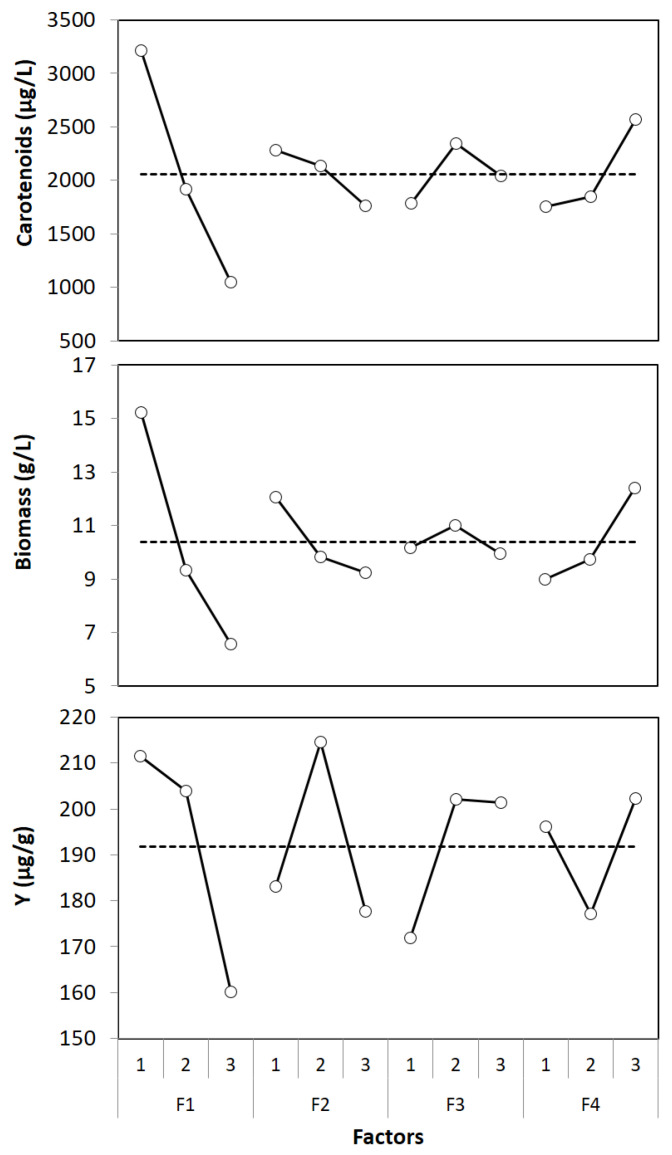
Individual influence of each factor evaluated on carotenoid production.

Urea composition presented the most significant impact on biomass growth and carotenoid production (75–77%), and in both responses, the second factor with the high effect is whey composition (12–13%). Meanwhile, ethanol and pH explain a small fraction (9.7–9.8%) of the results achieved in carotenoid and biomass growth in the range of evaluated settings. As for the yield, again, nitrogen composition was the factor with a higher effect. However, the effect was lower than for the other two responses (39.3%). Additionally, for this response, the second effect was the utilized pH (20.3%). The average effects are displayed in Figure 1 to obtain insight into each factor’s individual effects at the studied levels. 

The factor with the highest effect on all responses (urea composition) showed almost the same trend. A negative linear effect was obtained; that is, the production and growth were indirectly proportional to urea’s utilized concentration. Although the ability of *Rhodotorula glutinis* to express urease activity for catalyzing the hydrolysis of urea has been reported [37], a high concentration could negatively affect microorganism growth and the production of carotenoids. 

As for the effect of the concentration of whey, the effect is opposite to that of nitrogen concentration. Both carotenoids and biomass increased by increasing the carbon source concentration. Nevertheless, a high initial glucose content has been related to carotenoid synthesis inhibition [38]. Surely, nitrogen and carbon utilized concentrations were the most influential parameters (87–90%) dominating the process performance in terms of carotenoid production and microorganism growth. From these results, we hypothesized that the C/N ratio strongly affects carotenoid production; an experimental evaluation would be required to validate this sole factor. However, by looking at the results of the data presented in Table 2 and because these results can mainly be attributed to the effect of carbon and nitrogen concentration, a clear increment can be observed in the production of carotenoids by increasing the C/N ratio at the three levels of whey concentration utilized. Moreover, optimum levels were obtained at the higher high carbon source concentration and lower nitrogen concentration, that is, a C/N of 17:1.

Braunwald et al. (2013) tested different ratios between carbon and nitrogen (ammonium sulfate) and reported that carotenoid production was increased (1.247 mg/L of total carotenoids) with the highest ratios between carbon and nitrogen sources (120:1). Otherwise, at lower ratios (20:1), carotenoid production was the lowest (0.097) mg/L of total carotenoids. Conversely, in another study using ammonium sulfate as the nitrogen source, a C/N ratio of 10:1 was reported to increase the volumetric production of carotenoids by *Rhodotorula glutinis*. When urea has been utilized, a direct positive correlation between carotenoid production and C/N has been reported but with different optimum C/N ratios (31:1–45:1) [39].

Likewise, it has been found that *Rhodotorula glutinis* cultivated under high C/N ratios (70:1–100:1) promotes lipid accumulation with a reduced carotenoid production [40]. It is difficult to establish an optimum C/N from the literature, but there is a tendency to obtain high carotenoid biosynthesis with a high C/N ratio among almost all the cited literature.

Regarding the effect of pH and ethanol (factors with the least effect) for P and B, these factors presented a negative linear and quadratic effect, respectively. Regarding Y, the evaluated factors’ individual effects varied from those presented for P and B. Only the effect of urea and ethanol are similar; however, pH and the utilized carbon concentration presented a quadratic effect. 

Concerning the influence of ethanol, although it explains a small fraction of the total production of carotenoids (µg/L), its effect is significant for this response. Additionally, it was observed that the effect of ethanol among the evaluated levels was not significant (*p* < 0.05) on the obtained biomass but explained a high fraction of the carotenoids obtained per biomass (µg/g), which indicates that ethanol at a concentration below 2% *v*/*v* does not affect the microorganism growth but is related to the biosynthesis of carotenoids. Furthermore, the optimum carotenoid production was obtained at an intermediate level (1% *v*/*v*). This phenomenon has been stated in the literature; *Rhodotorula glutinis* tolerates ethanol at moderate concentrations (0.2–3.6% *v*/*v*), and also, its addition to the culture media stimulates the synthesis of carotenoids [41]. This effect has been related to the production of carotenoids by different microorganisms such as *Xanthophyllomyces dendrorhous*. Marcoleta et al. (2011) studied the effect of carbon source on gene expression in the yeast *Xanthophyllomyces dendrorhous* [42]. They reported that ethanol (2 g/L) caused the induction of the expression of *CrtYB* and *CrtS* genes and promoted the synthesis of carotenoids. This phenomenon could be expected because ethanol is not readily assimilated as a fermentable carbon source, but it directly affects the synthesis of carotenoids once the pathway starts.

As for the pH, it presented the lowest effect on carotenoid production among the evaluated factors, with an optimum pH for carotenoid production of 4.5 (lower level). Moreover, it can be noticed that the pH relative influence is higher for biomass (8.6%) and even higher for carotenoid production on biomass (20.3%). The individual effects displayed that both carotenoids and biomass were negatively affected by the increment of pH. Conversely, this factor presented a quadratic effect on the carotenoids per biomass with an optimum at an intermediate level. To some extent, this is obvious due to the negative contribution from pH 4.5 to 5.5 being higher for biomass than for total carotenoid production.

Other authors have studied the effect of pH on the carotenoid production of different strains. Valduga et al. (2008) tested different pH values (3–8) [43]. Additionally, they reported a reduction in carotenoid production in the *Sporidiobolus salmonicolor* strain by increasing the pH above 4 (optimum level in such a study), reaching 455.4 mg/L of total carotenoids [43]. On the other hand, for *Rhodotorula glutinis*, different optimum initial pH values have been reported from 3 to 7 [44,45]. Therefore, the optimum pH will depend on the utilized media, and so, it should be evaluated when a new media is being designed. 

If all interactions are assumed negligible, the optimum levels are the ones where the maximum production of carotenoids was obtained (target responses). These conditions were nitrogen source of 0.3% *w*/*v*, pH of 4.5, ethanol concentration of 1% *v*/*v*, and carbon source of 6% *w*/*v*. Optimal conditions, the contribution of each factor in such conditions, and the expected production of carotenoids are shown in Table 4. 

Expected production was 4230.30 µg/L (showing a total contribution of all factors of 2172.39 µg/L with a grand average performance of 2057.91 µg/L). The experimental design was validated by evaluating carotenoid production under the optimum performance obtaining a carotenoid production of 4075.00 µg/L, biomass of 18.6 g/L, and yield of carotenoids per biomass of 219.08 µg/g. The validation results for all responses were slightly lower than the predicted response; however, all validation results were within the estimated 90% confidence interval.

In bioprocesses for carotenoid production, few reports use the Taguchi methodology. The Taguchi method certainly allows for obtaining a considerable amount of information about the evaluated factors with minimum experimental requirements; however, it requires assuming that all interactions between factors are negligible. 

Shinde and Lele (2010) optimized the lutein production by the microalgae *Auxenochlorella protothecoides* using a Taguchi design to determine sucrose’s influence, yeast extract KH_2_PO_4_, Mg_2_SO_4_·7H_2_O, CaCl_2_·2H_2_O, EDTA, and pH. The maximum lutein production reached was 630.2 µg/L. Sucrose and yeast extract had a higher influence than other factors evaluated. The Taguchi method was recently applied for optimizing the component levels in a waste-based designed medium for the production of carotenoids by *Xanthophyllomyces dendrorhous* [14]. In such a study, it was proposed that if the validation results were within the expected confidence interval, then the optimum levels and the identified main effects could be accepted, and if not, there is a possibility that the interactions among the factors are significant. This study’s results obtained during the validation studies were within the expected confidence interval. Thus, the assumption of not having interacting effects among factors was accepted for the studied factors at the evaluated levels. 

The present study showed enhanced carotenoid production of 4075 µg/L, which was around 200% higher than the grand average obtained at all trials (Table 4). These results demonstrate the benefits that can be obtained with the Taguchi method. Likewise, it was shown that hydrolyzed goat milk whey is a promising source for carotenoid synthesis. Certainly, there are various types of dairy industry by-products from different animal sources that can serve as potential low-cost raw materials for the synthesis of added-value compounds such as carotenoids [46].

### 3.3. Carotenoid Identification

**Figure 2:**
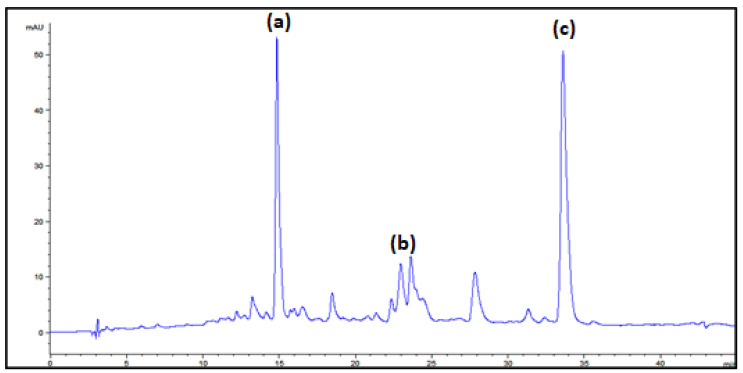
Carotenoid profile from *Rhodotorula glutinis* P4M422 cultivated in goat milk whey: (a) β –carotene, (b) γ –carotene and (c) torulene.

HPLC analysis was performed in order to identify the major individual carotenoids produced by *Rhodotorula glutinis* P4M422 in a medium based on goat milk whey. The results indicated that the qualitative profile was unaffected, although some quantitative differences were observed in some trials. The major carotenoids produced were the provitamin A carotenoids β–carotene, γ–carotene and torulene. Invariably, β–carotene and torulene levels were higher relative to those of γ–carotene. A typical chromatogram corresponding to trial 2 is shown in Figure 2. 

According to the results, the different medium compositions evaluated did not significantly affect the qualitative profile of carotenoids. However, some variations in the proportions of the carotenoids produced were observed between some trials. β- and γ-carotene were readily identified by comparing their spectroscopic and chromatographic characteristics with those of standards. Torulene, a carotene biosynthesized by *Rhodotorula* sp. and other yeasts exhibiting distinctive spectroscopic features, was identified by comparing such characteristics with those reported elsewhere [34]. 

**Figure 3:**
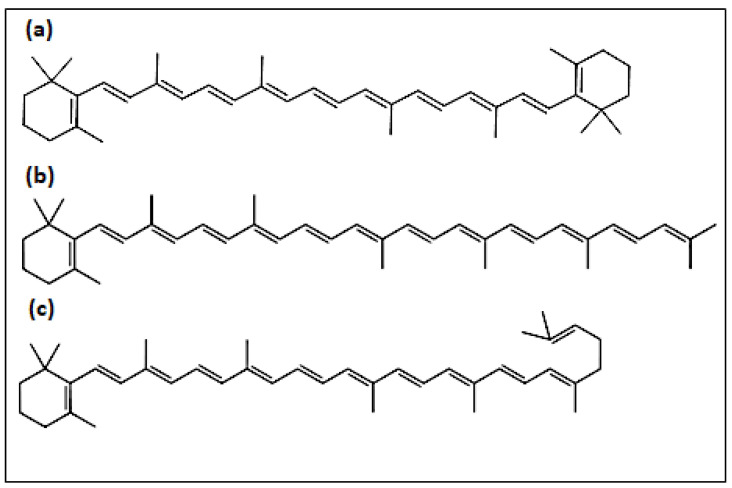
Chemical structure of main carotenoids produced by *R. glutinis* P4M422: (**a**) β-carotene, (**b**) torulene, and (**c**) γ-carotene.

Table 5 summarizes the retention times and spectroscopic features of the carotenoids identified. β-carotene (Figure 3a), containing 11 conjugated double bonds and 2 rings, was the carotenoid that eluted earlier, followed by γ-carotene (11 conjugated double bonds and only a terminal ring, Figure 3c). The levels of the latter compound were invariably the lowest. It was observed that several geometrical isomers of this compound might be formed, a subject that deserves further investigation in future studies. Torulene (13 conjugated double bonds and only 1 terminal ring, Figure 3b) was the latest eluting carotene. This was invariably the major carotenoid in all the trials. Having an unsubstituted β-ring and the appropriate unsaturated backbone that share provitamin A carotenoids [4], the study of the bioavailability and metabolism of torulene in humans appears as an interesting topic from a nutritional point of view. 

On the other hand, having 13 c.d.b., it absorbs maximally at 484 nm (Table 5), a wavelength considerably longer than common food carotenoids [47]. Considering this previous study [47], such absorption maxima would correspond to a distinctive reddish color among carotenoids, which can be of interest in the agro-food industry as colorant for foods or feeds. 

The circular use of resources, the harnessing of untapped carotenoids, and their microbial sources are a timely topic in the context of sustainable health promotion and can lead to innovations in the relevant industry for the development of diverse products, including functional foods, nutraceuticals, nutricosmetics, or novel foods, among others [45]. 

## 4. Conclusions

Hydrolyzed goat milk whey showed promising expectations as a low-cost medium for provitamin A carotenoid production by *Rhodotorula glutinis* P4M422. Medium components and pH were evaluated through the Taguchi method. The carbon and nitrogen composition had the most significant influence on the process performance. Under improved conditions, carotenoid production (4075 µg/L) was enhanced by almost 200%. The study demonstrates the utility of applying a statistical design such as the Taguchi method for assessing the factors’ impact on a bioprocess and maximizing the desired response.

The identified carotenoids were torulene, β-carotene, and γ-carotene. Results are important for the innovative sustainable production of carotenoid-rich matrices, featuring provitamin A carotenoids that can be nutritionally important to tackle vitamin A deficiency. Indeed, the study of new carotenoid sources, production approaches, and properties of little-studied carotenoids is a timely topic in the carotenoid field as highlighted by the Ibero-American Network for the Study of Carotenoids as Functional Foods Ingredients (IBERCAROT; http://www.cyted.org/?q=es/detalle_proyecto&un=829 (accessed on 12 December 2022) and the European Network to Advance Carotenoid Research and Applications in Agro-Food and Health (EUROCAROTEN; http://www.eurocaroten.eu (accessed on 11 December 2022), https://www.cost.eu/actions/CA15136/#tabs|Name:overviews (accessed on13 December 2022)).

## Figures and Tables

**Table 1:** Independent variable (factors) on medium composition based on GMW and their levels.

Factors	Levels		
	1	2	3
Urea (F1, % *w*/*v*)	0.3	0.5	0.7
pH (F2)	4.5	5.5	6.5
Ethanol (F3, % *v*/*v*)	0.1	1.0	1.9
Glucose (F4, % *w*/*v*)	3.5	4.5	6.0

**Table 2:** Taguchi L9 orthogonal array and total carotenoid, biomass, and yield results at each trial.

Run	F1	F2	F3	F4	Total Carotenoids (µg/L)	Biomass (g/L)	Yield (µg/g)
1	1	1	1	1	2860.6 ± 111.1	15.3 ± 1.4	187.2 ± 10.5
2	1	2	2	2	3369.7 ± 147.0	14.6 ± 0.2	229.7 ± 8.8
3	1	3	3	3	3406.1 ± 75.7	15.6 ± 0.2	217.5 ± 8.1
4	2	1	2	3	2933.3 ± 111.1	13.6 ± 1.0	215.9 ± 24.7
5	2	2	3	1	1672.7 ± 109.1	7.0 ± 0.5	240.5 ± 32.8
6	2	3	1	2	1139.4 ± 137.7	7.3 ± 0.7	155.2 ± 4.5
7	3	1	3	2	1042.4 ± 105.0	7.1 ± 1.1	146.3 ± 8.2
8	3	2	1	3	1357.6 ± 42.0	7.8 ± 0.2	173.5 ± 10.3
9	3	3	2	1	739.4 ± 42.0	4.6 ±0.5	160.6 ± 27.2

**Table 3:** Analysis of variance (ANOVA) results for total carotenoids, biomass, and yield.

Factors	Variance			F-Ratio			Relative Influence (%)		
	TC	B	Y	TC	B	Y	TC	B	Y
F1	10,690,223.4	176.3	6903.4	987.7	268.1	21.6	76.9	75.4	39.3
F2	640,195.9	20.0	3563.8	59.1	30.4	11.1	4.6	8.6	20.3
F3	711,747.8	2.8	2661.9	65.8	4.2	8.3	5.1	1.2	15.2
F4	1,759,608.2	28.7	1553.1	162.6	43.6	4.9	12.7	12.3	8.8
Error	10,823.4	0.7	319.8				0.7	2.5	16.4
Total	1,069,168.1	18.0	1350.8				76.9	75.4	39.3

**Table 4:** Optimum levels and contribution of factors and expected results for the validation trial.

Factors	Optimum Levels	Contribution		
		P (µg/L)	B (g/L)	Yx (µg/g)
F1	1	1154.2	4.9	19.6
F2	1	220.9	1.7	-8.7
F3	2	289.6	0.6	10.2
F4	3	507.7	2.0	10.5
Current grand average of performance		2057.9	10.4	191.8
Contribution of all factors		2172.4	9.2	31.6
Expected result		4230.3	19.6	223.5
Confidence interval		154.2	0.8	17.2

**Table 5:** Optimum levels and contribution of factors and expected results for the validation trial.

Peak	Assigned Carotenoid	*t*_R_/min	λ_max_ ^a^	λ_max_ ^b^
a	β-carotene	14.7	426, 450, 476	425, 450, 477
b	γ-carotene	22.7	438, 460, 490	437, 462, 494
c	torulene	33.1	460, 486, 518	460, 484, 520

Wavelengths of maximum absorption of ^a^ carotenoids produced by *Rhodotorula glutinis* P4M422 and ^b^ reported in the literature [34].

## Data Availability

Data is contained within the article.
